# Genome-Wide Linkage Disequilibrium and the Extent of Effective Population Sizes in Six Chinese Goat Populations Using a 50K Single Nucleotide Polymorphism Panel

**DOI:** 10.3390/ani9060350

**Published:** 2019-06-13

**Authors:** Haile Berihulay, Rabiul Islam, Lin Jiang, Yuehui Ma

**Affiliations:** Institute of Animal Science, Chinese Academy of Agricultural Sciences (CAAS), Beijing 100193, China; haile.berihulay@yahoo.com (H.B.); md.rabiul27@yahoo.com (R.I.)

**Keywords:** goat, effective population size, linkage disequilibrium, minor allele frequency, SNP

## Abstract

**Simple Summary:**

Information on linkage disequilibrium (LD) and the extent of effective population size (Ne) has important implications for exploring the degree of biological diversity, for predicting underlying selection pressure, and for designing animal breeding programs. In this study, we assessed LD, Ne, and the distribution of minor allele frequency in six goat populations. Accordingly, the results of LD and Ne using a single nucleotide polymorphism (SNP) panel (Caprine SNP 50K BeadChip, Lincoln, NE, USA) are helpful for the sustainable conservation, proper management, and utilization of Chinese goat populations.

**Abstract:**

Genome-wide linkage disequilibrium is a useful parameter to study quantitative trait locus (QTL) mapping and genetic selection. In many genomic methodologies, effective population size is an important genetic parameter because of its relationship to the loss of genetic variation, increases in inbreeding, the accumulation of mutations, and the effectiveness of selection. In this study, a total of 193 individuals were genotyped to assess the extent of LD and Ne in six Chinese goat populations using the SNP 50K BeadChip. Across the determined autosomal chromosomes, we found an average of 0.02 and 0.23 for r^2^ and D’ values, respectively. The average r^2^ between all the populations varied little and ranged from 0.055 r^2^ for the Jining Grey to 0.128 r^2^ for the Guangfeng, with an overall mean of 0.083. Across the 29 autosomal chromosomes, minor allele frequency (MAF) was highest on chromosome 1 (0.321) and lowest on chromosome 25 (0.309), with an average MAF of 0.317, and showing the lowest (25.5% for Louping) and highest (28.8% for Qingeda) SNP proportions at MAF values > 0.3. The inbreeding coefficient ranged from 0.064 to 0.085, with a mean of 0.075 for all the autosomes. The Jining Grey and Qingeda populations showed higher Ne estimates, highlighting that these animals could have been influenced by artificial selection. Furthermore, a declining recent Ne was distinguished for the Arbas Cashmere and Guangfeng populations, and their estimated values were closer to 64 and 95, respectively, 13 generations ago, which indicates that these breeds were exposed to strong selection. This study provides an insight into valuable genetic information and will open up the opportunity for further genomic selection analysis of Chinese goat populations.

## 1. Introduction

Goats (*Capra hircus*) are animals that are highly adaptable to diverse environmental conditions, are raised all over the world for meat, milk, skin, fibre, and manure production, and are the sole source of livelihood for many rural people and landless laborers [1,2]. Although they present reasonable reproductive and productive performance, it is necessary to improve their production efficiency to become more competitive with other livestock industries. In this regard, genetic selection plays a vital role and substantial genetic gain has been achieved using traditional breeding methods.

The commercial application of genomic selection implies that relatively large numbers of important breeding animals are genotyped using dense marker covering [3], which opens up the possibility of performing quantitative trait loci (QTL) mapping studies with greater power and precision. In genomic studies, identifying traits of interest and genomic polymorphisms are required. In most whole genome marker data sets, the causal variants are usually not included but the effects of quantitative loci are reflected by markers that are in linkage disequilibrium (LD) with the causal loci [4]. Determining the extent and decay of LD within a population will provide new opportunities to determine the number of markers that are required for uncovering the genetic basis of relevant traits in livestock populations [5]. Most researchers define LD as a nonrandom association between alleles of different loci in a given population, used in order to achieve high-accuracy marker-assisted selection and to reduce breeding strategy errors [6]. The pattern of LD between adjacent markers is generally high, decreases with increasing marker distance, and is affected by various factors, including genetic drift, population growth/structure, mutation, artificial/natural selection, and the recombination rate. LD can be variable among populations and loci [6]. LD could be the most widely important measurement of connectedness between allele pairs and haplotype block structures across a given population [7].

The presence of LD patterns and the extent of Ne are important population genetic parameters that have recently received a great deal of research attention [8], determining population demographic development [9] and demographic processes such as migration and admixture [10], and having profound implications for understanding the architecture of the animal genome [6,11]. The Ne is widely regarded as one of the most critical population parameters because it measures the rates of genetic drift and inbreeding and affects the efficacy of systematic evolutionary forces such as mutation, selection, and migration [12]. It also helps to discover population demographic history, and allows one to predict the behavior of genetic diversity through time. The Ne is estimated using the r^2^ coefficient and measures the observed range and the amount of genetic variation within a frame of population genetics. It also provides information on the degree of inbreeding of the population under consideration [13]. The Ne determines the amount of genetic variation, genetic drift, and linkage disequilibrium (LD) in populations [6].

Accordingly, LD and Ne provide substantial genomic selection accuracy in goat breeding [14]. In recent decades, several studies have demonstrated the usefulness of LD and Ne features by applying advanced technologies, such as the single nucleotide polymorphism (SNP) Chip panel and whole genome sequencing in various livestock species including goat [14], cattle [13], sheep [6,8], and pig [15]. The application of genomic SNP will provide an opportunity to identify a high resolution of LD and to understand the effectiveness of selection in an individual breed. However, information about LD and Ne using SNP 50K data in Chinese goat populations is generally unexploited. China is a big country with complex geographical borders (Qinling Mountains, Huaihe River Line), Plateaus, river systems, a dynamic geological history, and diverse domestic goat breeds; these goat breeds exhibit enormous genetic variation in terms of production, reproduction, hypoxia, and heat tolerance performance. Therefore, the objective of this study was to characterize genome-wide LD and the extent of Ne in six Chinese goat populations using the Caprine SNP50 BeadChip, which is essential for conservation and for designing new breeding strategies.

## 2. Materials and Methods

All animal work was conducted according to the guidelines for the care and use of experimental animal survival established by the Ministry of Agriculture of China and given by the animal ethics committee of Institute of Animal Science, Chinese Academy of Agricultural Sciences (IAS-CAAS-AE-03).

### 2.1. Animals and Sampling Units

The dataset included the genotypes of 193 animals representing the Nanjiang (*n* = 23), Qingeda (*n* = 24), Arbas Cashmere (*n* = 58), Jining Grey (*n* = 39), Louping (*n* = 24), and Guangfeng (*n* = 24) breeds. These breeds are mainly found in five Chinese provinces (Table 1).

**Figure 1:**
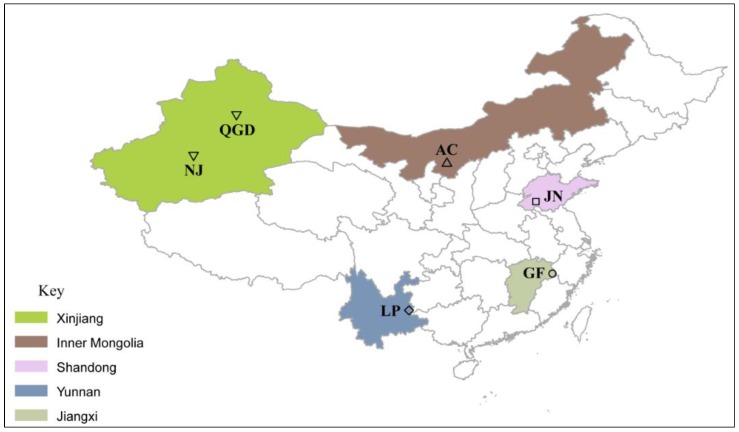
Geographic distribution and provinces of the six Chinese goat populations.

To minimize the likelihood of studying related individuals, samples were collected from different areas to capture a representative sample of within-breed genetic diversity. The geographic distribution of Chinese breeds sampled in this study is depicted in Figure 1.

Whole blood samples were used to extract genomic DNA. Blood samples of approximately 10 mL were drawn out from the jugular vein into a tube. Extracted DNA was put in the safe area and stored at −20 °C and/or at 4 °C. DNA extraction was conducted at the laboratory of the Institute of Animal Science, Chinese Academy of Agricultural Sciences, using Promega Kits according to the manufacturer’s procedures. The DNA concentrations for all samples were measured using a Nano-DNA spectrophotometer (ND-1000), considering the A_260_/A_280_ absorbance ratio. The quantity and quality of genomic DNA were measured using 1% agarose gel electrophoresis (Merck & Co., Kenilworth, NJ, USA) and a Qubit^®^ 3.0 fluorometer (Thermo Fisher Scientific, Waltham, MA, USA).

### 2.2. Genotyping and Quality Control

All DNA samples were genotyped using the Illumina goat SNP 50K Bead Chip (Illumina, San Diego, CA, USA) containing 53,347 single nucleotide polymorphisms (SNPs). To ensure data quality, quality control (QC) was performed across the six goat populations by removing any SNPs with call rate <95%, MAF <0.05 and Hardy–Weinberg equilibrium (HWE) (*p* < 10^−5^) using PLINK v. 1.07 [16]. Moreover, samples with more than 10% missing genotypes were removed from the data set. Finally, SNPs that had high linkage disequilibrium (LD) were pruned using the indep-pairwise command parameters (SNP window size: 50, SNPs shifted per step: 5, r^2^ thresholds: 0.2) by the PLINK software, as recommended in the PLINK manual [16]; this left 39,552 SNPs for further analysis. Pruning is essential to produce a better comparison between populations because some stretches of SNPs have a low MAF.

### 2.3. Population Analyses

To assess the population stratification of the data, we carried out principal component analysis (PCA) implemented in PLINK v1.09 [16]. Additionally, level of admixture was analyzed through a model-based clustering using ADMIXTURE v.1.3 [17]. After CV errors were estimated for each K-value, the K-value with the lowest CV error was chosen as optimal. Neighbor-joining tree was constructed based on Nei’s genetic distance between pairwise individuals and were performed by PowerMarker v3.25 [18].

### 2.4. Minor Allele Frequncy and Inbreeding Coefficient

PLINK v1.07 [16] was used for the estimation of minor allele frequency (MAF) under the default settings [16] for all autosomal SNPs. The distribution of MAF in each population was represented as a proportion of all the SNPs used in the analysis, and then five categories were established, representing the proportion of SNPs with MAF values that fell within the following ranges: 0.0–0.1, 0.1–0.2, 0.2–0.3, 0.3–0.4, and 0.4–0.5. The results were plotted for comparison between all the populations, using Microsoft Excel. The inbreeding coefficient was calculated as a function of the expected and observed homozygote difference, using the following equation:

(1) $$F_{IS}=\frac{\left(O_{i}-E_{i}\right)}{\left(L_{i}-E_{i}\right)}$$

where $F_{IS}$ is the assessed inbreeding coefficient of the $i^{th}$ animal, $O_{i}$ is the number of homozygous loci observed in the $i^{th}$ animal, $E_{i}$ is the number of homozygous loci expected and $L_{i}$ is the number of genotyped autosomal loci [16].

### 2.5. Linkage Disequilibrium (LD) Analysis

The standard descriptive LD parameters r^2^ and D’ were estimated as previously described by [19] and [20], respectively. LD is the squared correlation coefficient between the two loci [21]. The average r^2^ and D’ values were calculated for each chromosome via Haploview software [19]. The LD was estimated genome-wide for each breed, respectively. The decay of LD (r^2^) values were then sorted by inter-SNP distance and binned into different inter-SNP intervals (0–10 kb, 10–20 kb, 20–40 kb, 40–60 kb, 60–80 kb, 80–100 kb, 100–200 kb, 200–500 kb, 500 kb–1 Mb, 1–2 Mb, and >2 Mb), followed by an analysis of mean LD within each bin.

### 2.6. Effective Population Size (Ne)

Effective population size (Ne) was estimated based on the following equation: $Ne=\frac{1}{4c}\left(\frac{1}{E\left(r^{2}\right)}-1\right)$ [22], where Ne is the effective population size, c is the genetic distance in morgans, and $E\left(r^{2}\right)$ is the expected $r^{2}$ for distance c, implemented in the software SNeP v1.1 [23]. Time points representing the number of generations ago (T) was calculated as: $T=\frac{1}{2c}$ [24]. Ne was estimated for each chromosome and generation in the past.

## 3. Results

### 3.1. Descriptive Statistics

**Figure 2:**
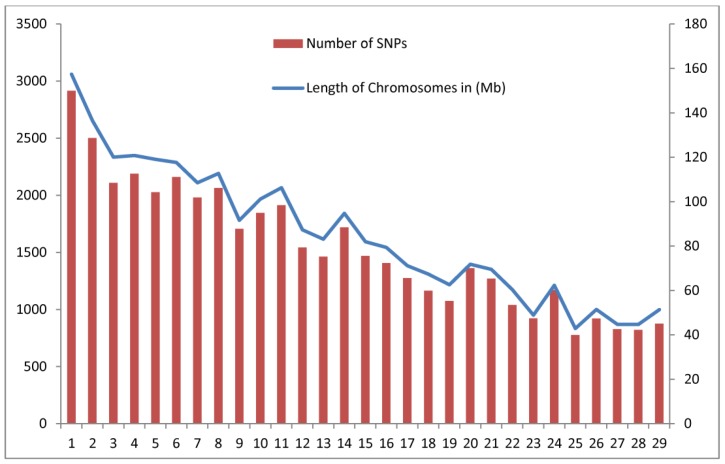
Summary of SNPs and chromosome lengths included in the analysis. Red bars indicate the number of SNPs and the blue line indicates chromosome length.

In the present study, 53,347 SNPs were used before quality control. About 5946 SNPs were removed, leaving 47,401 of the loci distributed over 29 autosomal chromosomes, which were used for downstream analysis. About 2663 SNPs were removed due to having a MAF of <0.05. About 1568 and 1715 SNPs were removed on the basis of their results for Hardy–Weinberg Equilibrium (<10^−5^) and call rate (<0.95), respectively. About 7849 SNPs were removed using LD-based pruning when an r^2^ threshold of 0.2 was exceeded. After filtering, 39,552 SNPs (74.14%) were used for LD analysis. The SNPs spanned approximately 0.43 GB of the caprine autosomal genome. The total SNP numbers per each chromosome (CHI) are summarized in Figure 2, ranging from 777 for CHI 25 to 2916 for CHI 1. The total chromosome length was 2466.19 Mb, with an average of 85.04 Mb. Chromosome 1 was found longer and chromosome 25 was shortest with 157.4 Mb and 42.86 Mb, respectively. The average distance between marker pairs for this analysis was 259.22 kb, with the distance between markers ranging from 257.51 kb to 260.25 kb.

### 3.2. Population Genetic Structure and Admixture

**Figure 3:**
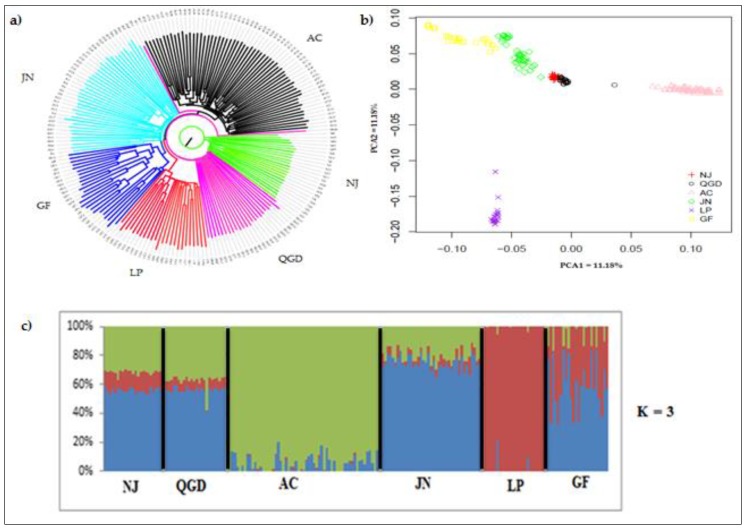
Population genetic structure and admixture analysis of six Chinese goat populations: (**a**) Neighbor-joining (NJ) tree. (**b**) Principal component analysis. (**c**) Level of admixture among the six goat breeds [25].

A neighbor-joining (NJ) tree clustering method represents relationships based on Nei’s genetic distance. The NJ tree showed four major clades: the Louping and Arbas Cashmere goats clustered separately at distinct clades (Figure 3a). The Qingeda and Nanjiang goats clustered together in one clade, while Jining Grey and Guangfeng goat breeds form one group in the other clades. Similarly to the results of the NJ tree, principal component analysis (PCA) showed four different clusters (Figure 3b) corresponding to their origin. Principal components analysis 1 and 2 together accounted for 19.81% of the total genetic variation. These components clearly revealed four different clusters, which corresponded to their genetic distance and relatedness between populations. The first principal component explained 11.18% of the observed global variation and divided the samples into three clusters. Qingeda and Nanjiang breeds were cloth clustered and Jining Grey and Guangfeng breeds were also grouped together, while the Louping breeds were separately positioned. The second principal component accounted for 8.63% of the total variance and separated Arbas Cashmere independently. To provide additional insight into the genetic variation and admixture of the studied goat breeds, ADMIXTURE software was used to conduct model-based clustering of all individuals. Based on the lowest cross validations error, K = 3 was the optimal number of ancestral populations and separated the populations corresponding to their origin (Figure 3c). This analysis revealed a high level of admixture in the NJ, QGD, and GF populations, whereas the Arbas Cashmere, Louping, and Jining Grey breeds revealed relatively low levels of admixture, which is in accordance with the clustered results shown by PCA and the neighbor-joining tree. The admixture revealed the six breeds according to their geographical distribution and historic origins.

### 3.3. Minor Allele Frequency, Linkage Disequilibrium, and Inbreeding Coefficient

In the current study, the mean MAFs for all the autosomal SNPs were 0.276 (AC), 0.308 (QGD), 0.284 (NJ), 0.314 (JN), 0.255 (LP), and 0.258 (GF), as indicated in Table 2. The distributions of MAF across the populations are presented in Figure S1. The LP (26.5%) showed the highest proportion of SNPs in the lowest MAF interval compared to the proportion of SNPs observed in the higher MAF intervals. The LP and QGD goats showed the lowest and highest SNPs proportions in the MAF >0.3 intervals. The proportion of SNPs for the AC, JN, and QGD populations was found to be higher with increasing MAF interval, and showed a decrease at the last interval. However, no clear pattern was observed for the GF and NJ populations.

**Figure 4:**
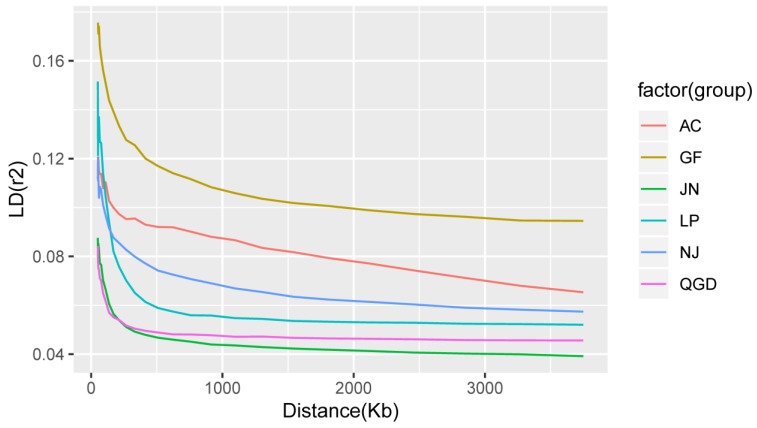
LD decay (r^2^) from 0 up to 3000 kb for the six Chinese goat breeds.

The descriptive statistical results for each chromosome of LD between adjacent markers and the F_IS_ are shown in Table 3. The average mean r^2^ and D′ values estimated between adjacent SNPs for the 29 autosomal chromosomes were 0.02 and 0.23, respectively. The average r^2^ results were lowest for the JN group (0.055) and highest for the GF group (0.128), with an overall mean of 0.083. The r^2^ measure is known to be a more robust parameter for bi-allelic marker analysis, and therefore requires a smaller sample size for accurate LD estimation than D’ [26]. The average LD (r^2^) decreased with genetic distance for all groups (Figure 4). The observed values of r^2^ for all the populations, with the SNPs for each bin separated by intervals of 0–10 kb, 10–20 kb, 20–40 kb, 40–60 kb, 60–80 kb, 80–100 kb, 100–200 kb, 200–500 kb, 500 kb–1 Mb, 1–2 Mb, and >2 Mb are presented in Table S1.

LD, r^2^, and D’ were estimated on a per-chromosome basis and are summarized in Table 3. Moreover, as shown in Figure 4 and Table S1, the mean existing r^2^ decreased more slowly with increasing physical distance between SNPs (the values continued to decline, just more slowly). The mean LD (r^2^) showed the most rapid decline in the first five bins. Overall, the JN, QGD, and NJ goat breeds displayed the lowest LD values across all intervals as compared to AC, GF, and LP populations. For each of the 29 autosomal chromosomes, the inbreeding coefficient ranged from 0.064 to 0.085 with an average of 0.075.

### 3.4. Effective Population Size (Ne)

**Figure 5:**
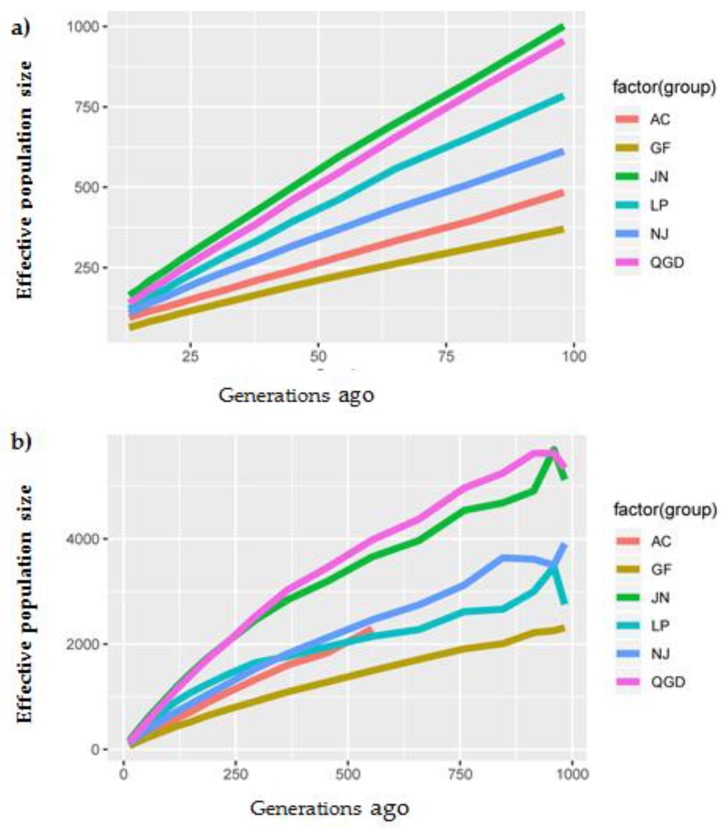
Average estimated effective population sizes in six goat populations. (**a**) Ne in the past 100 generations. (**b**) Ne over the past 1000 generations.

Historical and recent effective population sizes for the studied goat populations are presented in Figure 5. Figure 5a shows that the historical *Ne* declined between 1000 and 100 generations ago across the six studied goat populations. In total, the estimated Ne had decreased in AC (from 2289 to 95), NJ (from 3908 to 109), (JN from 5695 to 164), LP (from 3469 to 121), QGD (from 5630 to 140), and GF (from 2308 to 64) at 13 generations ago, which reflected a downward trend in Ne for all breeds due to selection. A rapidly decreasing recent Ne was observed in the GF and AC, while the JN and QGD had a slow Ne decline trend (Figure 5b).

## 4. Discussion

In our previous study, we revealed the genetic diversity and population structure of goat populations [25]. Considering this fact, here, we estimated genome-wide linkage disequilibrium and the extent of effective population size in six Chinese goat populations using a 50K SNP panel. The measures of LD demonstrate some allele frequency dependence in the finite sample sizes [27]. The MAF is important because LD, independent of the metric used, is a function of allelic frequency. A low MAF may correspond to a larger difference in the allele frequency of coupled alleles, which can result in lower estimates of LD as measured by either r^2^ or D’ [28]. Accordingly, filtering QC criteria and thresholds can affect the distribution and extent of LD [29], since there is an important association between high levels of LD and a higher proportion of SNPs with high MAF values. Across the 29 autosomal chromosomes, MAF was highest on chromosome 1 (0.321) and lowest on chromosome 25 (0.309), with an average MAF of 0.317, and showing the lowest (25.5% for LP) and highest SNP proportion (28.8% for QGD) at MAF values of >0.3. These results are comparable with the values reported for different Ethiopian goats [30] and Sahiwal cattle types [31]. However, our study found values higher than the reported MAF values of 0.27 (Drakensberger), 0.26 (Bonsmara), 0.28 (Angus), and 0.28 (Holstein) cattle [32]. The inbreeding coefficient measures the percent of increase in homozygous gene pairs in an individual relative to the average of the breed [33]. In the current study, across the autosomal chromosomes, the inbreeding coefficient ranged from 0.064 to 0.085 with an average mean of 0.075. These results are similar to the previous report of F_IS_ = 0.04 for Brazilian Santa Inês sheep [11], but are lower than the 0.29 value found in various South African cattle breeds [32].

LD varies among populations, genomic regions, and between pairs of markers in close proximity [34]. The declining pattern of linkage disequilibrium in this population is consistent with those reported by previous studies in different breeds of cattle [32] and buffalo [9]. The r^2^ and D’ measures are important descriptive LD parameters [35]. The r^2^ is a more robust measure of LD because it is less sensitive to allele frequency and a small sample size. The average r^2^ between all the studied populations varied little and ranged from 0.055 for JN to 0.128 for GF, with an overall mean of 0.083. The r^2^ values obtained in the current study were higher than the reported values of 0.11 for different South African, French, and Argentinian Angora goats [36], and the values ranged from 0.14 to 0.17 for South African commercial goats [37], and from 0.14 to 0.24 for different Canadian and Australian goat breeds [38]. We observed that Chromosome 1 was the longest and chromosome 25 was the shortest. Similar results were previously reported in different cattle breeds [39,40]. The lowest r^2^ observed between breeds and chromosomes might be due to differences in genetic drift, recombination rates, and less intensive selection which resulted in a lower LD across the genome [41]. Generally, a declining pattern of average r^2^ values was observed when moving from 10 to 200 kb. Consistent with this study, a decreasing trend was previously reported for goats [36], sheep [6], cattle [12], and pigs [42], with existing r^2^ remaining higher than approximately 0.4 for a distance up to 60–80 Kb. Furthermore, D’ is a good measure to clarify the extent of LD in a population and the variation in LD over the genome [43]. We estimated the average D’ value at 0.23 across the 29 chromosomes. These results were lower than the previously reported values of 0.48 for various South African commercial goats [37], 0.55 for Sahiwal cattle [31], and the value ranged from 0.12 to 0.52 for Nellore cattle [40], which could be due to the higher power for detecting LD when using markers with many alleles.

Effective population size (Ne) is a crucial population genetic parameter because of its relationship to the loss of genetic variation, increases in inbreeding, the accumulation of mutations, and the determination of the accuracy of genomic selection [3]. The current study observed a decreasing trend in Ne from 1000 to 100 generations ago across the six studied populations. Interestingly, the JN, QGD, NJ, and LP breeds showed higher Ne estimates after 13 generations ago, suggesting that these animals could have been affected by selection. The rapid increase pattern in Ne may also include bottlenecks associated with domestication, selection and breed formation, and the endangerment of the breed [12]. Therefore, it is useful to consider our results in the context of the demographic history of the goat population in China. This finding is consistent with our previous study of genetic diversity and population structure, in which it was discussed that the principal component and admixture analysis clearly clustered the population corresponding to their geographic distribution and genetic construction [25]. Comparatively, a decreasing recent Ne was observed for the AC and GF breeds, and their estimated values were closer to 64 and 95 at 13 generations ago, suggesting that these animals were strongly affected by selection pressure or genetic drift which resulted in population decline [44]. The AC goat breeds are among the most well-known Cashmere goat breeds, which have experienced strong artificial selection for the production of a high Cashmere yield [45]. They are an important breed for livestock husbandry in China due to their excellent cashmere traits such as coarse wool, hair, softness, slender texture, and good gloss [46]. Otherwise, it is well known that a small Ne means the reduction of genetic variation in the population, thereby hindering genetic progress. The Ne slope in Figure 4b suggested that the AC and GF population sizes were consistently decreasing, signifying that actions are needed to maintain sufficiently large Ne, such as the reduction of the widespread use of artificial insemination, which introduces new bloodlines from exotic breeds, as well as smaller progeny groups for elite sires and an increase in recorded goat buck numbers. Consistent with our study, Ne values of about 67, 57, and 93 were reported for Argentinian, French, and South African goat populations at ten generations ago [36]. Furthermore, a study by [14] revealed that the ecotype goat was slightly higher in effective population sizes than the Tankwa and commercial breeds across generations.

## 5. Conclusions

In this study, we found low to moderate LD among the six populations, which indicates that the Caprine SNP 50K BeadChip will likely be a feasible tool for the prediction of genomic breeding values in goat populations. From the result showing a decline in LD with genetic distance, we inferred the Ne values and showed a rapidly decreasing trend across the entire studied population. Moreover, this study revealed a decreasing trend of Ne with the estimated values closer to 64 for AC and 95 for QGD at 13 generations ago, implying that the decline in the Ne of these goat breeds should be avoided. Further confirmatory investigations for the current population are required on a larger population dataset.

## Figures and Tables

**Table 1:** Summary of breed name, sample code, sample size, and physical distribution of six Chinese indigenous goat populations.

Breed Name	Sample Code	Sample Size (*n*)	Province
Nanjiang	NJ	23	Xinjiang
Qingeda	QGD	24	Xinjiang
Arbas Cashmere	AC	59	Inner Mongolia
Jining Grey	JN	39	Shandong
Louping	LP	24	Yunnan
Guangfeng	GF	24	Jiangxi

**Table 2:** Summary of MAF in the six goat populations.

Breed Name	Code	MAF < 0.05
Nanjiang	NJ	0.284
Qingeda	QGD	0.308
Arbas Cashmere	AC	0.276
Jining Grey	JN	0.416
Louping	LP	0.353
Guangfeng	GF	0.366

**Table 3:** Summary of analyzed markers and average linkage disequilibrium (r2 and D’) separated by different distances across the 29 autosomal chromosomes.

CHR	SNP (*n*)	Size (Mb)	D’	r^2^	F_IS_	MAF	Distance (Mb)
1	2916	157.4	0.225	0.023	0.072	0.324	0.260
2	2502	136.51	0.225	0.023	0.075	0.327	0.260
3	2109	120.04	0.233	0.022	0.079	0.316	0.259
4	2190	120.74	0.229	0.023	0.064	0.319	0.259
5	2027	119.02	0.229	0.022	0.072	0.318	0.259
6	2161	117.64	0.235	0.023	0.073	0.318	0.259
7	1981	108.43	0.237	0.024	0.069	0.320	0.260
8	2064	112.67	0.226	0.022	0.085	0.321	0.259
9	1706	91.57	0.234	0.022	0.073	0.315	0.259
10	1846	101.09	0.223	0.021	0.079	0.319	0.259
11	1914	106.23	0.234	0.023	0.073	0.314	0.259
12	1543	87.28	0.229	0.023	0.073	0.318	0.259
13	1463	83.03	0.228	0.022	0.074	0.313	0.259
14	1719	94.67	0.231	0.022	0.080	0.312	0.260
15	1470	81.90	0.227	0.022	0.068	0.312	0.260
16	1406	79.37	0.237	0.024	0.083	0.312	0.260
17	1275	71.14	0.234	0.023	0.081	0.315	0.259
18	1165	67.28	0.223	0.021	0.070	0.318	0.259
19	1074	62.52	0.229	0.023	0.078	0.318	0.260
20	1362	71.78	0.224	0.022	0.072	0.320	0.259
21	1271	69.43	0.225	0.022	0.084	0.317	0.258
22	1040	60.28	0.231	0.023	0.077	0.317	0.259
23	922	48.87	0.212	0.020	0.064	0.321	0.259
24	1171	62.31	0.239	0.024	0.070	0.317	0.260
25	777	42.86	0.232	0.021	0.077	0.309	0.258
26	920	51.42	0.223	0.022	0.070	0.324	0.258
27	828	44.71	0.218	0.020	0.084	0.319	0.259
28	822	44.67	0.224	0.022	0.082	0.316	0.260
29	876	51.33	0.227	0.023	0.074	0.316	0.260

## Supplementary Materials

The following are available online at https://www.mdpi.com/2076-2615/9/6/350/s1, Figure S1: Distribution of minor allele frequency (MAF) for each studied population. The percentage of SNP is plotted for each frequency bin, Table S1: The average observed values of r^2^ were calculated for each bin in each inter-SNP distance class.

## Data Accessibility

The data on genome-wide linkage disequilibrium and the extent of effective population sizes in the six Chinese goat populations used in this paper are deposited and available at https://www.animalgenome.org/repository/pub/CAAS2018.1121/.
