# Supplementary material for: Genome-Wide Linkage Disequilibrium and the Extent of Effective Population Sizes in Six Chinese Goat Populations Using a 50K Single Nucleotide Polymorphism Panel
Source: Animals (Basel). 2019 Jun 13;9(6):350. doi: 10.3390/ani9060350 (PMC6617254; doi:10.3390/ani9060350)
Supplement: Supplementary file 1 [file animals-09-00350-s001.pdf]

Supplementary Table1. The average observed values of r^2 were calculated for each bin in each inter-SNP distance class

bin	QG	NJ	LP	JN	GF	AC
0-10KB	0.56+0.33	0.59+0.31	0.62+0.34	0.66+0.33	0.63+0.33	0.73+0.31
10-20 KB	0.50+0.21	0.53+0.23	0.59+0.29	0.48+0.20	0.55+0.29	0.51+0.25
20-40 KB	0.40+0.20	0.42+0.21	0.49+0.25	0.41+0.20	0.47+0.23	0.43+0.22
40-60 KB	0.37+0.19	0.39+0.19	0.45+0.23	0.38+0.19	0.46+0.22	0.41+0.20
60-80 KB	0.35+0.17	0.37+0.17	0.43+0.22	0.36+0.17	0.43+0.20	0.38+0.18
80-100 KB	0.33+0.16	0.35±0.15	0.40+0.21	0.34+0.16	0.42+0.19	0.37+0.17
100-200 KB	0.30+0.12	0.33±0.13	0.37+0.18	0.31+0.16	0.39+0.18	0.35+0.14
200-500 KB	0.28+0.08	0.33±0.12	0.33+0.15	0.28+0.13	0.37+0.16	0.33+0.12
0.5 -1 Mb	0.28+0.04	0.32±0.11	0.32+0.14	0.29+0.09	0.37+0.13	0.33+0.12
1-2 Mb	0.28+0.02	0.31+0.11	0.31+0.12	0.27+0.08	0.36+0.15	0.30+0.11
>2 Mb	0.24+0.02	0.31+0.09	0.31+0.07	0.27+0.04	0.35+0.12	0.30+0.08
Average	0.35+0.15	0.39+0.15	0.42+0.20	0.37+0.14	0.44+0.18	0.40+0.18

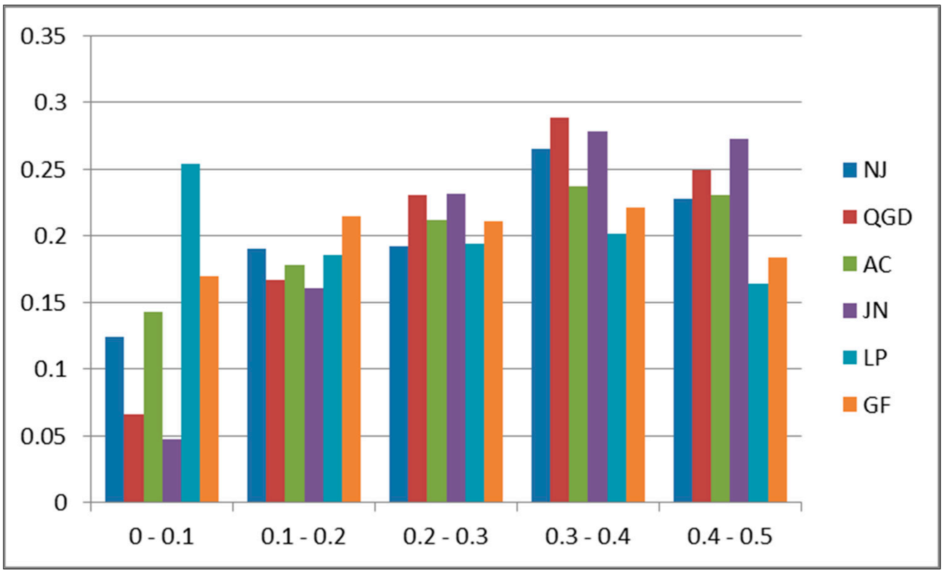

Figure S1 Distribution of minor allele frequency (MAF) for each studied population. The percentage of SNP is plotted for each frequency bin.
